# Pequi Fruit Extract Increases Antioxidant Enzymes and Reduces Oxidants in Human Coronary Artery Endothelial Cells

**DOI:** 10.3390/antiox11030474

**Published:** 2022-02-28

**Authors:** Karla M. S. Braga, Eugenio G. Araujo, Frank W. Sellke, M. Ruhul Abid

**Affiliations:** 1 Division of Cardiothoracic Surgery, Cardiovascular Research Center, Rhode Island Hospital, Warren Alpert Medical School of Brown University, Providence, RI 02903, USA; karla_da_silva_braga@brown.edu (K.M.S.B.); frank_sellke@brown.edu (F.W.S.); 2 School of Veterinary Medicine, Federal University of Goias, Goiania 74690-900, Brazil; earaujo@ufg.br

**Keywords:** human coronary artery endothelium, cardiovascular disease, ethnopharmacology, phenols, reactive oxygen species

## Abstract

Reactive oxygen species (ROS) imbalance results in endothelial cell function impairment. Natural phenolic antioxidant compounds have been investigated as therapeutic alternatives. The fruit bark of Brazilian-native pequi (*Caryocar brasiliense*, Camb.) is rich in polyphenols. The HPLC-MS (High-Performance Liquid Chromatography coupled with Mass Spectrometry) analyses identified gallic acid and catechin in six out of seven ethanolic extract samples prepared in our lab. In this study, we examined the effects of ethanolic pequi extract on ROS levels in human coronary artery endothelial cells (HCAEC) subjected to hypoxia or oxidative stress. We first confirmed the oxidant scavenging capacity of the extract. Then, HCAEC pre-incubated with 10 or 25 μg/mL of extract were subjected to hypoxia for 48 h or 100 μM H_2_O_2_ for six hours and compared to the normoxia group. Total and mitochondrial ROS levels and cell proliferation were measured. Pequi significantly reduced cytosolic HCAEC ROS levels in all conditions. Mitochondrial ROS were also reduced, except in hypoxia with 10 μg/mL of extract. HCAEC proliferation increased when treated with 25 μg/mL extract under hypoxia and after H_2_O_2_ addition. Additionally, pequi upregulated oxidative stress defense enzymes superoxide dismutase (SOD-)1, SOD-2, catalase, and glutathione peroxidase. Together, these findings demonstrate that pequi bark extract increases antioxidative enzyme levels, decreases ROS, and favors HACEC proliferation, pointing to a protective effect against oxidative stress.

## 1. Introduction

Oxidative stress (OS) is a deleterious condition present in major cardiovascular diseases (CVD), the leading cause of morbidity and mortality worldwide [1]. The Centers for Disease Control and Prevention (CDC) information confirmed that CVD accounted for 23% of deaths between 2016 and 2017 in the United States [2]. In CVD, endogenous or exogenous imbalance of reactive oxygen species (ROS) and insufficient production of antioxidant defenses [3] favor cell impairment and death. In addition, increased intracellular production of ROS in the vascular system relates to ischemic heart disease (IHD), along with endothelial cell (EC) malfunction [4].

Widely considered as alternatives to prevention and treatment of IHD, flavonoids are bioactive phenolic phytochemicals found in fruits and vegetables, with an apparent beneficial effect on the cardiovascular system related to antioxidant capacity through inhibiting endothelial NADPH oxidase and controlling nitric oxide levels in the vascular endothelium via the inhibition of superoxide synthesis [5]. Phenols are known for their antioxidant potential and could protect cells in acute CVD [6] and reduce risk in chronic conditions [7]. However, the failure of clinical trials using antioxidants in patients with IHD [8,9,10,11,12] challenges the prevailing view that ROS production is damaging to the microvasculature.

Pequi (*Caryocar brasiliense*, Camb.) is a native plant of central Brazil with a high concentration of phenols in its tough fruit bark (mesocarp and epicarp) [13]. Pequi is considered one of the prime plant species of the Brazilian savanna-like ecosystem, “Cerrado”, due to the importance of the fruit in regional cuisine, the extraction of oils for cosmetics, and therapeutic properties [14]. The epicarp flour and external mesocarp of pequi (bark) are rich in total dietary fiber and carbohydrates, ashes, magnesium, calcium, manganese, and copper [15]. Additionally, locals employ this vegetable in traditional medicine against influenza, colds, inflammatory diseases, wound healing, gastric lesions, menstrual dysfunction, ophthalmologic, hepatic, and even tumor control conditions [16]. 

More recently, pequi has been reported as analgesic and anti-inflammatory [17], ameliorating aging-related anemia, inflammation, and oxidative stress in Swiss mice [18], in addition to presenting anticholinesterase and antioxidant activities, along with the prevention of mice memory impairment from aluminum intake and brain lipid peroxidation [19]. Furthermore, others have shown that the ethanolic extract of pequi bark has very low toxicity in vitro [20] and in vivo [21], supporting its therapeutic potential. 

Despite the evident potential, knowledge is scarce about the antioxidant effects of pequi bark, particularly the mesocarp, as an alternative for cardiovascular protection against oxidative damage in cardiovascular system diseases. This research aimed to evaluate pequi bark’s ethanolic extract efficacy in protecting human coronary artery endothelial cells (HCAEC) subjected to OS or conditions that result in increased ROS production.

## 2. Materials and Methods

### 2.1. Pequi Extract Preparation

**Figure 1:**
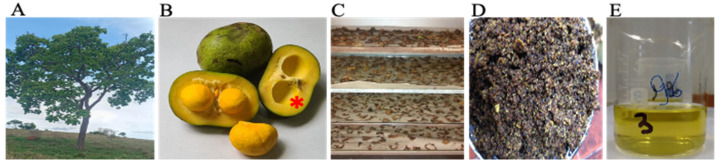
Pequi (*Caryocar brasiliense*, Camb.). The tree (left) is native to the Brazilian Cerrado (**A**), a savanna-like biome, with marked dry and wet seasons. The fruit (**B**) is edible and oily and has a thick, dense mesocarp and epicarp (asterisk) that form the bark, which is dried (**C**), macerated (**D**), and percolated in 95% ethanol to extract phenols. After percolation, the ethanol is removed by rotative evaporation (40 °C) and the final product (**E**) is obtained.

Pequi (Figure 1) barks (mesocarps) were harvested in central Brazil (15.032232″ S and 49.942103″ W at 730.5 m altitude). The botanical material was collected in a region of well-drained red soil, on a sunny day, and an exsiccate was deposited in the herbarium of the Federal University of Goias (Goiania, Brazil) under the number UFG—43-833. The barks were crushed to powder and dried. The pulverized material was subjected to cold maceration using 95% (*w*/*v*) ethanol as the extracting liquid (1:3). After maceration, filtration, and subsequent concentration in a rotary evaporator at 40 °C, we stored the final ethanol-free product (ethanolic extract of pequi bark) at −20 °C, protected from light.

### 2.2. Total Phenol Quantification and Antioxidant Activity In Vitro of Pequi Extract

Phenolic compounds were quantified by spectrophotometry at 700 nm using Folin–Ciocalteau phenol reagent (Sigma, Richmond, VA, USA), and the results are expressed in mg of gallic acid equivalent (GAE)/100 g of the sample reagent [22]. 

We used DPPH (2,2-diphenyl-1-picrylhydrazyl, Sigma^®^, Richmond, VA, USA) to access antioxidant activity in vitro. The degree of discoloration of the DPPH radical was measured spectrophotometrically in the aqueous solution, with a 0.2 mg mL^−1^ concentration. We also used Trolox, a Vitamin E analogue, as standard to calculate pequi extract’s antioxidant activity. A control sample with no added extract was also analyzed, and the scavenging percentage was calculated according to the following equation: DPPH scavenging capacity (%) = [A control − A sample/A control] × 100. A = absorbance at 520 nm.

### 2.3. Qualitative High-Performance Liquid Chromatography Coupled with High-Resolution Mass Spectrometry (HPLC-HRMS)

We prepared the samples with HPLC-grade methanol at a concentration of 200 ppm. The Thermo Scientific (Waltham, MA, USA) Ultimate 3000^TM^ liquid chromatograph with an ACE—C18 column (4.6 × 100 mm; 3 µm) coupled to the Thermo Scientific Q-Exactive^TM^ high-resolution mass spectrometer operated at H-ESI source, 4 kV spray voltage negative mode, 30 units/sheath gas, ten units/auxiliary gas, 350 °C capillary temperature, 300 °C auxiliary gas temperature, 55 tube lens, and 150–700 *m*/*z* mass range. We performed HPLC analysis with 0.1% acidified deionized water with formic acid (mobile phase A, *v*/*v*) and methanol acidified with 0.1% formic acid (mobile phase B—*v*/*v*). The gradient programming started with 93:07 (A: B%), 70:30 (A: B%) in 10 min; 50:50 (A: B%) in 5 min; 30:70 (A: B%) in 3 min; 20:80 (A: B%) in 2 min; and 100 (B%) in 3 min, remaining for 3 min. The run time was 26 min, with 0.3 mL/min flow rate, 10 µL injection volume, and 20 °C column temperature. We used a parallel reaction monitoring (PRM) experiment with collision energies equal to 30 for the fragmentation study. The analysis to identify phenolic compounds used a stock solution with standard phenolic compounds in methanol at a concentration of 1 mg.mL^−1^. From the stock solutions, we prepared the standard mix solution at a concentration of 50 µg mL^−1^ and performed the analysis of the standards under the same conditions as the sample. The phenolic compound standards used were gallic acid, protocatechuic acid, gentisic acid, caffeic acid, p-coumaric acid, vanillic acid, ellagic acid, catechin, epicatechin, rutin, quercetin, naringenin, luteolin, and kaempferol. We used Xcalibur™ software (version 4.2, Thermo Fischer Scientific, Waltham, MA, USA) to process the data. Seven samples of the pequi extract were analyzed.

### 2.4. Cell Culture

A cryopreserved vial of human coronary artery endothelial cells (Cat. No. CC-2585), containing ≥500,000 cells, was purchased from Lonza (Walkersville, MD, USA). Cells were maintained in Endothelial Cell Basal Medium-2 (EBM™-2, Cat. # 3156, Lonza, Walkersville, MD, USA) and supplemented with a Microvascular Endothelial Cell Medium-2 EGM™-2 MV SingleQuotes™ Kit (Cat. # CC-4147, Lonza, Walkersville, MD, USA) and 5% fetal bovine serum (Lonza, Walkersville, MD, USA). HCAEC were cultured under sterile conditions in 100 × 20 mm tissue culture dishes and maintained in a humidified atmosphere (37 °C ± 1 °C, 5% CO_2_, 90% ± 2%). Cells were fed three times weekly and subcultivated using trypsinization according to supplier’s instructions. Passages of 3–5 cells were used for further experiments.

### 2.5. Stress Induction in HCAEC

We performed 6 to 8 independent experiments for each condition (normoxia, hypoxia, H_2_O_2_, with or without pequi, etc.) with at least five replicates per condition. We formed groups of six units (each unit consisting of five 35 mm cell culture dishes) for each treatment, according to the stress induction strategy, hypoxia, or H_2_O_2_ exposure, to increase ROS production by HCAEC. When confluent, cells were trypsinized to form a cell suspension normalized to 1 × 10^4^ cells/mL, transferred to 96-well plates (100 μL per well), and pre-incubated with extract with 10 μg/mL, 25 μg/mL, or without extract (vehicle control) for 24 h. 

To induce hypoxia, we placed 96-well cell culture plates in a modular incubator chamber [23] (Hypoxia Chamber, MIC-101, Billups-Rothenberg, San Diego, CA, USA). Twin cell culture plates were prepared, one placed in the hypoxic chamber and the other maintained in normoxia. To create a hypoxia environment, we removed the oxygen by allowing a 95% N2 and 5% CO_2_ gas mixture into the chamber by opening the gas tank at a 20 L per minute flow rate. After seven minutes, we shut down the gas flow and sealed the chamber by closing the clamps. The chamber was then placed in a conventional incubator for 48 h at 37 °C. 

We also induced stress by incubating HCAEC by adding 100 μM H_2_O_2_ to each well for six hours. 

### 2.6. Determination of Cytosolic ROS Production in HCAEC

Cytosolic ROS levels were measured in HCAEC as follows: 25 μM 2,7-dichlorodihydrofluorescein diacetate (H2DCF-DA) fluorescent probe (Sigma-Aldrich, St. Louis, MO, USA) were added to HCAEC at 37 °C, in the dark, and incubated for 30 min. In the presence of ROS, the cell-permeable nonfluorescent H2DCF-DA turns into the fluorescent 2′,7′-dichlorofluorescein (DCF). Fluorescence intensity levels at excitation and emission wavelengths of 485 and 528 nm, respectively, were measured in a microplate reader. We normalized optical densities (OD) of experimental samples by subtracting OD values of respective background wells without DCF reagent. 

### 2.7. Determination of Mitochondrial ROS Production in HCAEC

HCAEC (1 × 10^4^ cells/well) were grown as previously described, except that we formed groups of eight units (each unit consisting of five 35 mm cell culture dishes). MitoSox^TM^ Red reagent (Invitrogen, Carlsbad, CA, USA) solution was freshly prepared by adding dimethyl sulfoxide (DMSO) to a MitoSox vial (500 μM). Modified HBSS (Hank’s Balanced Salt Solution, JRH Biosciences, Lenexa, KS, USA) was mixed with MitoSox reagent as described in the manufacturer’s protocol. Here, 100 μL of the mix was added to each well with HCAEC and incubated at 37 °C for 15 min. The absorbance was measured at 510 nm to excitation and 580 nm to emission. Optical densities were normalized by the subtraction of respective background wells without MitoSox reagent.

### 2.8. Proliferation Assay in HCAEC Subjected to Hypoxia and H_2_O_2_

HCAEC were detached from plates by enzymatic dissociation using trypsin–ethylenediaminetetraacetic acid (EDTA) to form a cell suspension normalized to 6 × 10^4^ cells/mL. Here, 10,000 HCAEC/well were plated to a 96-well plate and pre-incubated with extract with 10 μg/mL, 25 μg/mL, or without extract (vehicle control, DMSO) for 24 h; each group consisted of six wells. We induced OS in HCAEC by adding 100 μM of H_2_O_2_, except in controls, to five groups of six repetitions each for six hours. We subjected another group of cells to hypoxia in a chamber with 95% N_2_ and 5% CO_2_ for seven minutes, followed by 48 h inside a humidified incubator at 37 °C with 5% CO_2_, while maintaining control cells under normoxia. Nuclear-stained diamidino-2-phenylindole (DAPI) was applied to each well at 1 μg/mL and discarded after 15 min. Cells were observed on a fluorescence microscope and scanned images were analyzed using Image J software (version 1.53, NIH, Bethesda, MD, USA). 

### 2.9. Western Blotting

HCAEC were grown as described above to 80–90% confluence. DMSO (Pequi Extract vehicle) was added to controls, whereas pequi extract (25 μg/mL) was added to the media for 48 h. Cell lysates were prepared and Western Blot (WB) analyses were performed as previously described [24]. We used primary antibodies against SOD-1 (catalogue #37385T), SOD-2 (#13141S), catalase (#12980S) GPX-1 (#3206S), SirT-1 (#9475T) p-GSK3ß (#5558P), LC3A/B (#4108), and PGC1-alpha (#NBP1-04676SS). All antibodies were purchased from Cell Signaling (Danvers, MA, USA), except PGC1-alpha (from Novus Biologicals, Centennial, CO, USA).

Quantitative densitometric analysis of the WB was carried out using NIH Image J (version 1.53, NIH, Bethesda, MD, USA). Protein bands were normalized against glyceraldehyde 3-phosphate dehydrogenase (GAPDH) or alpha tubulin.

### 2.10. Statistical Analysis

For the cell proliferation studies, averages of five images of each well were converted to arbitrary units, considering the means of the respective controls as 100. Each treatment was compared to the respective control and the results were subjected to Mann–Whitney’s test.

For ROS measurements, results from each experimental group were compared to respective controls using Mann–Whitney’s test. For WB analysis, band densitometries of pequi-extract-treated groups and respective controls were compared using Student’s t-test. 

All analyses were performed with Prism^®^ software (Version 9.3.1, GraphPad Software, San Diego, CA, USA), considering *p* < 0.05.

## 3. Results

### 3.1. Pequi Extract Is Rich in Phenolic Compounds

**Figure 2:**
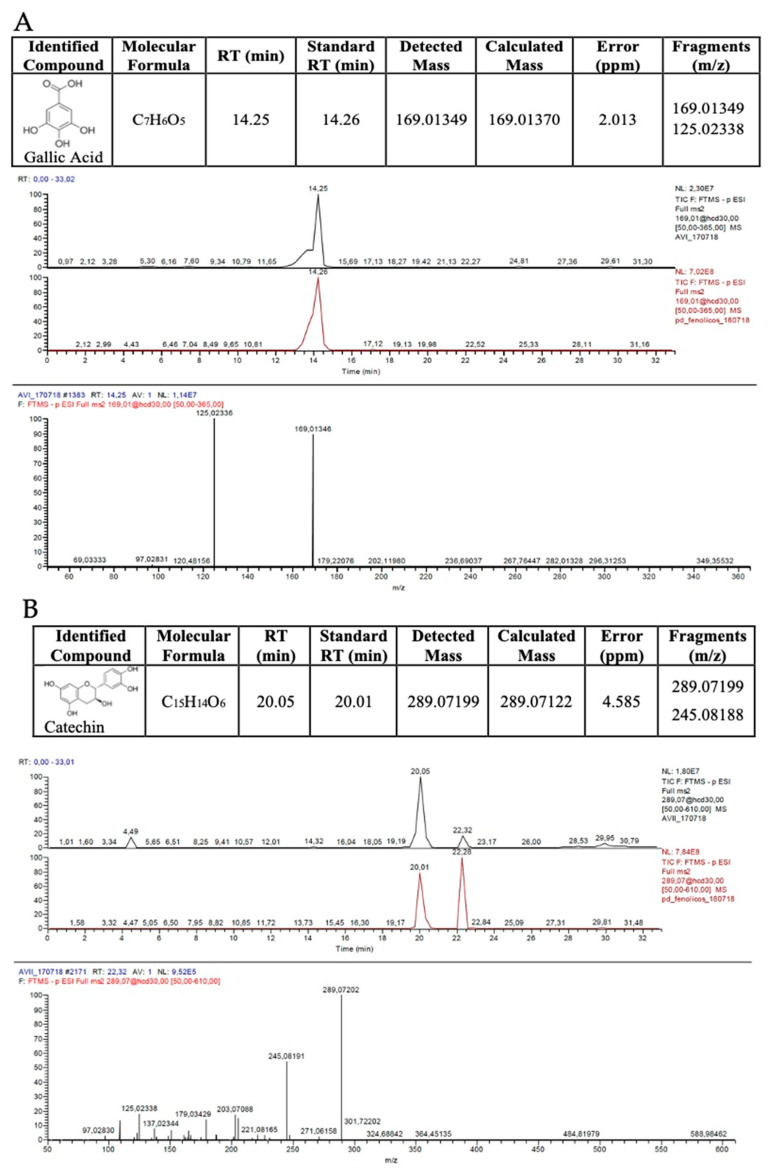
Qualitative high-efficiency liquid chromatography coupled to high-resolution mass spectrometry of the ethanolic extract of pequi bark. Representative chromatographic profiles of components detected in six of seven samples: (**A**) gallic acid; (**B**) catechin. RT—retention time.

The qualitative (HPLC-HRMS) analysis showed that the pequi extract contained gallic, protocatechuic, gentisic, caffeic, p-coumaric, vanillic, and ellagic acids, as well as catechin, quercetine, epicatechin, rutin, naringenin, luteolin, and kaempferol. HPLC profiles of gallic acid and catechin, the common compounds present in six of seven samples analyzed, are depicted in Figure 2. 

### 3.2. Pequi Extract Scavenges ROS In Vitro

**Figure 3:**
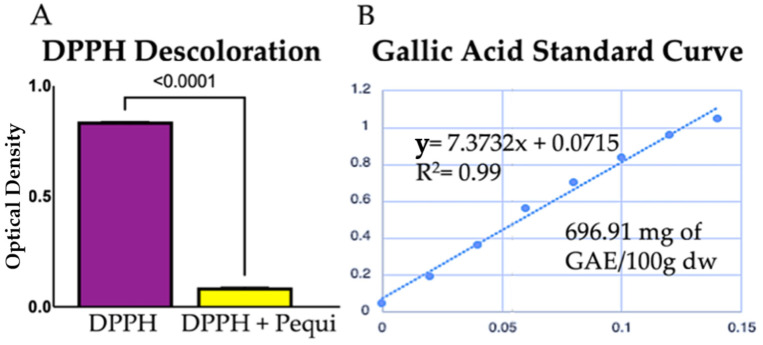
Pequi bark ethanolic extract is rich in phenolic compounds and scavenges free radicals in vitro: (**A**) graphic representation of DPPH reagent discoloration (92.62 ± 0.23%) as a measure for free radical neutralization by pequi extract; (**B**) gallic acid standard curve and concentration of total phenol in the extract based on gallic acid equivalence are shown. DPPH: 2,2-diphenyl-1-picrylhydrazyl.; GAE: gallic acid equivalent; dw: dry weight.

Our data demonstrated that the total phenol concentration in the pequi extract was 696.91 mg of GAE/100 g of dry weight (dw). To examine the free radical scavenging (neutralization) capacity of pequi extract, we performed the DPPH discoloration assay as described in the Materials and Methods. As a result, pequi extract demonstrated significant free radical scavenging potential, as shown by the 92.61 ± 0.23% rate of DPPH discoloration (Figure 3). In addition, radical neutralization of pequi extract potential reached 30.1 ± 1.27 μM of Trolox/g dw, further confirming the radical scavenging potential of the extract.

### 3.3. Pequi Extract Can Reduce Cytosolic and Mitochondrial ROS Levels in HCAEC

**Figure 4:**
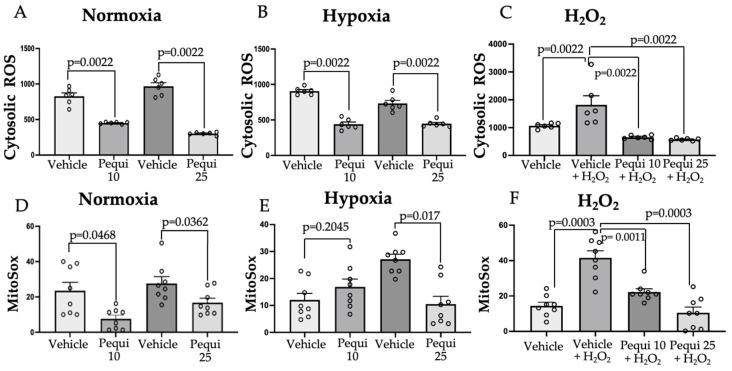
Cytosolic (**A**–**C**) and mitochondrial (**D**–**F**) Reactive Oxygen Species (ROS) production in human coronary artery endothelial cells (HCAEC) subjected to normoxia, hypoxia, and H_2_O_2_. HCAEC (1 × 10^4^ cells/well) were pre-treated with 0 (vehicle control), 10, or 25 μg/mL of pequi extract for 24 h, followed by 48 h of normoxia (**A**,**D**), hypoxia (**B**,**E**), or six-hour exposure to 100 μM of H_2_O_2_ (**C**,**F**). Pequi extract decreased cytosolic ROS production in all treatments and concentrations as compared to controls (*p* < 0.05). The trend was similar for mitochondrial ROS, except for the pequi 10 μg/mL treatment in hypoxia conditions, where mitochondrial ROS were not significantly different from vehicle control without extract. Vehicle = Dimethyl sulfoxide (DMSO); Pequi 10 = 10 μg/mL; Pequi 25 = 25 μg/mL. Statistical analyses were performed using Mann–Whitney’s test. The results shown represent *n* = 6 (**A**–**C**) or *n* = 8 (**D**–**F**) independent experiments, using 5 replicates for each experiment/condition. MitoSox: Mitochondrial Superoxide Indicator.

Pequi extract (10 and 25 μg/mL) reduced cytosolic ROS production in HCAEC (Figure 4A–C). The reduction was observed in both hypoxia for 48 h (39 ± 6% for 25 μg/mL, Figure 4B) and H_2_O_2_ exposure for six hours (68.46 ± 17.87% for 25 μg/mL, Figure 4C). Interestingly, the same effect was registered in HCAEC under normoxia for 48 h (45 ± 5.7%, Figure 4A). Although both extract concentrations of the extract reduced ROS, the effect was more prominent in the 25 μg/mL group. Noticeably, the extract reduced cytosolic ROS in all conditions.

Similarly, pequi extract significantly reduced mitochondrial ROS levels in all conditions (Figure 4D–F), except for hypoxia, where HCAEC were treated with 10 μg/mL of pequi (Figure 4E). For the groups treated with 25 μg/mL of pequi extract, mitochondrial ROS levels were reduced by 64.29 ± 6% in hypoxia (Figure 4E), by 74.7 ± 12.53% in HCAEC treated with H_2_O_2_ (Figure 4F), and by 39.4 ± 16.7% in normoxia (Figure 4D).

### 3.4. Pequi Extract Increases Proliferation in HCAEC

**Figure 5:**
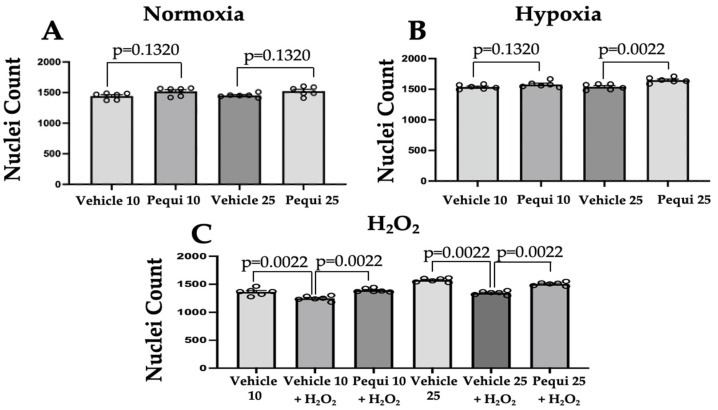
Human coronary artery endothelial cell proliferation assay. HCAEC were pre-treated for 24 h with 0 (Control), 10, or 25 μg/mL of Pequi extract. Cells (1 × 10^4^ cells/well) were subjected to normoxia (**A**) or hypoxia (**B**) for 48 h or 100 μM H_2_O_2_ for six hours (**C**). Cell proliferation significantly increased in hypoxia (25 μg/mL group) as compared to vehicle-only (DMSO) control. Although proliferation decreased after addition of H_2_O_2_ in HCAEC without extract, an increase in proliferation was observed when cells were pre-treated with pequi extract. Variance analysis revealed differences between the treatments (**C**). Experiments were carried out using six (*n* = 6) independent experiments, each consisting of 5 replicates per condition/group. Statistical analysis was carried out using Mann–Whitney’s test.

The addition of pequi extract (10 and 25 μg/mL) under normoxia conditions did not significantly increase HCAEC proliferation as compared to vehicle (DMSO) control (Figure 5A). In contrast, in HCAEC under hypoxia, proliferation significantly increased (by 5 ± 2.15%; *p* = 0.0022) when treated with 25 μg/mL of pequi extract, but not with 10 μg/mL (*p* = 0.1320) (Figure 5B).

In contrast, when we added H_2_O_2_ (100 μM) to the media for six hours, HCAEC proliferation was reduced significantly by 14.58 ± 1.27%. (Figure 5C). The addition of 10 μg/mL of extract increased proliferation by 10.34 ± 1.63% (*p* = 0.0022) as compared to vehicle control with H_2_O_2_ (Figure 5C). Likewise, the addition of 25 μg/mL of the extract also increased proliferation by 10.74 ± 1.28% (*p* = 0.0022).

### 3.5. Pequi Extract Induces Expression of Antioxidant Enzymes in HCAEC

**Figure 6:**
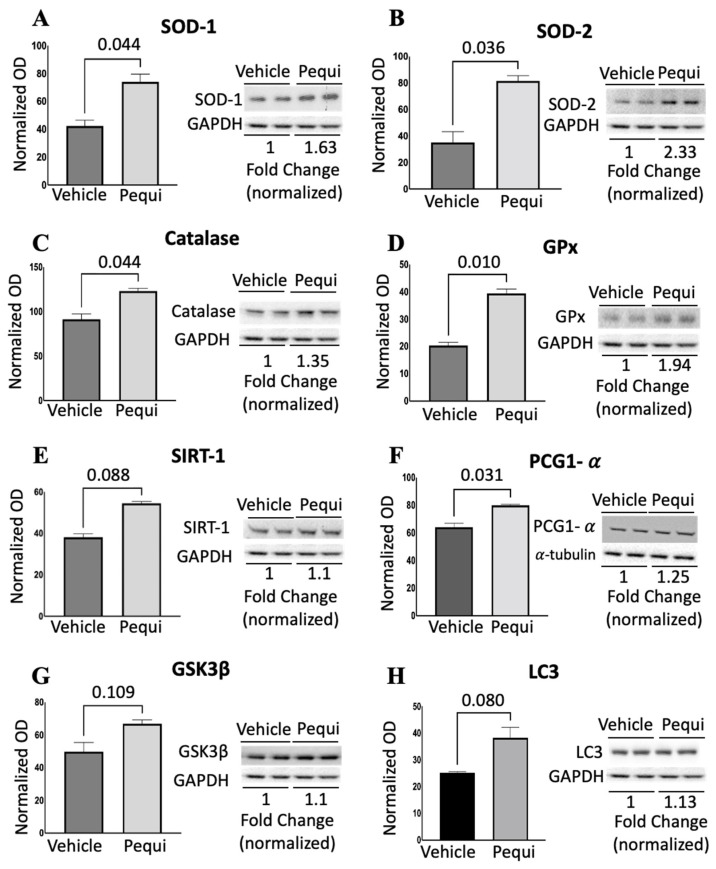
Pequi extract increases expression of antioxidant enzymes in HCAEC. Cells were cultured, pre-treated with pequi extract (25 μg/mL) for 24 h under normoxia conditions, and total protein content was extracted for Western blots analysis for Superoxide dismutase (SOD)-1 (**A**), SOD-2 (**B**), catalase (**C**), glutathione peroxidase—GPx (**D**), sirtuin 1—SIRT-1 (**E**), peroxisome-proliferator-activated receptor gamma coactivator 1-alpha (PCG1-α) (**F**), glycogen synthase kinase 3 beta (GSK3β) (**G**), and microtubule-associated protein 1A/1B-light chain 3 (LC3) (**H**). Western blots were normalized using α-tubulin or glyceraldehyde 3-phosphate dehydrogenase (GAPDH) as loading control. Five independent Western blot experiments were carried for each condition. Representative images are shown here. Statistical analysis was performed using Student’s *t*-test (*p* < 0.05). OD: Optical Density.

To examine the effects of pequi extract on oxidant signaling pathways in HCAEC, we performed Western blot (WB) analysis. HCAEC were treated with 25 μg/mL of pequi extract for 24 h and subject to WB (Figure 6A–F). Superoxide dismutase 1 (SOD-1, cytosolic) and superoxide dismutase 2 (SOD-2, mitochondrial) enzymes were significantly upregulated in HCAEC treated with pequi compared to control. In addition, catalase, glutathione peroxidase (GPx), and peroxisome-proliferator-activated receptor gamma coactivator 1-alpha (PCG1-α) were also increased in HCAEC treated with pequi. On the other hand, sirtuin 1 (SIRT-1), glycogen synthase kinase 3 beta (GSK3β), and microtubule-associated protein 1A/1B-light chain 3 (LC3) expression levels were not affected (Figure 6E,G,H).

## 4. Discussion

The main finding of this work was that the ethanolic extract of pequi significantly increases the expression levels of several antioxidant enzymes, decreases cytosolic and mitochondrial ROS, and increases proliferation in HCAEC. In addition, ROS reduction in HCAEC was more prominent when cells were subject to oxidative stress using H_2_O_2_.

The antioxidant effect of pequi is likely to relate directly to the phenolic compounds identified in the extract. Such biomolecules have been widely associated with favorable effects related to their antioxidant capacity. For instance, flavonoids from plants inhibited cell death, ROS accumulation, mitochondrial membrane depolarization, and apoptosis during hypoxia or reperfusion injury [25].

The qualitative phenolic composition of our pequi extract, as analyzed by HPLC-MS, was very similar to the pattern present in the *Nymphaea nouchali* leaf extract, including gallic acid, catechin, epigallocatechin, epi-catechin gallate, caffeic acid, luteolin, and kaempferol. Furthermore, this *N. nouchali* extract showed potent free radical scavenging ability, protected cellular DNA damage, and decreased ROS production [26], features we observed with pequi extract. In fact, gallic acid and catechin were identified by electrospray ionization mass spectrometry in a previous study that used a pequi peel ethanolic extract [1]. Equally important, both gallic acid and catechin were also present in a pequi leaf extract obtained in the same region we obtained the pequi fruit for our extract and showed anticholinesterase and antioxidant activities in mice [19]. 

Pequi extract resulted in more than 90% discoloration in the DDPH reagent, demonstrating that the extract possesses significantly potent free radical scavenging capacity. Notably, the free radical scavenging percentage of pequi is higher than 25 of 26 medicinal plants reported for DPPH radical scavenging capacity [27]. In addition, Mexican medicinal plants *Jatropha dioica* (dragon’s blood), *Flourensia cemua* (far bush), *Eucalyptus camaldulensis* (Eucalyptus), and *Tumera diffusa* (Damiana), found in harsh semi-arid climates resembling the dry season of the Brazilian “Cerrado” showed lower DPPH scavenge activity [28] than the extract we used. Interestingly, pequi extract has also been reported to inhibit chemically induced iron peroxidation in rat liver microsomes [29].

Using the vitamin E analogue Trolox as standard to calculate the pequi extract antioxidant activity (30.1 μM of Trolox/g DW) allowed a comparison to similar investigations. For instance, the Trolox-equivalent antioxidant capacities of 62 fruits frequently consumed worldwide were lower than that of pequi, except for olive (80.68 ± 2.11 μM of Trolox/g DW) and pomegranate (40.61 ± 0.11 μM of Trolox/g DW). Nevertheless, the total phenolic content of pequi extract (696.91 mg of GAE/100 g DW) was higher than all those 62 fruits [30]. A point often overlooked is that most studies, such as the one previously referred to [30], use the edible parts of the fruit. In our study, we used an extract obtained from an inedible part of a Brazilian “Cerrado” plant that could develop as a pharmaceutical option for oxidative-stress-related diseases and a sustainable economic alternative to aid environmental conservation of a threatened, important biome.

The reduction in cytosolic ROS production in HCAEC was observed in hypoxia for 48 h and H_2_O_2_ exposure for six hours. Similarly, many studies have described ROS inhibition by phenol-rich plant extracts. For instance, *Myrica rubra* extract significantly attenuated the intracellular ROS levels induced in H9c2 cardiomyocytes subjected to six hours of hypoxia than to a normal medium to mimic reperfusion [25]. Furthermore, the etheric and methanolic extracts of the *Laserpitium krapffii* fruits contained phenolic compounds that demonstrated antioxidant capacity to inhibit oxidative damage in chronic degenerative diseases [31].

The decrease in mitochondrial ROS in endothelial cells was even more remarkable, and was observed in all tested conditions (normoxia, hypoxia, and H_2_O_2_) with 25 μg/mL pequi extract. We previously demonstrated that a long-term increase in cytosolic ROS resulted in nitrotyrosine-mediated inactivation of mitochondrial (mito) antioxidant MnSOD, resulting in an increase in mito-ROS, loss of mitochondrial membrane potential (ΔΨm) reduction in EC proliferation, and angiogenesis [24]. We report here that the addition of pequi extract reduced mitochondrial and cytosolic ROS and increased HCAEC proliferation, suggesting plausible long-term protection for the endothelium of critical organs such as the heart.

The mechanism of the findings reported in this study showing a significant reduction in oxidants by pequi extract requires further investigation. Referring to our previously reported “ROS paradox”, oxidants can have beneficial or detrimental effects on endothelial cells, depending on various factors such as the quantity, sub-cellular location, duration of ROS exposure, and surrounding environmental milieu [4]. Therefore, the decrease in ROS we have described in the current study may be involved in modulating redox and other signaling pathways and may relate to both physiological and pathological conditions [32].

**Figure 7:**
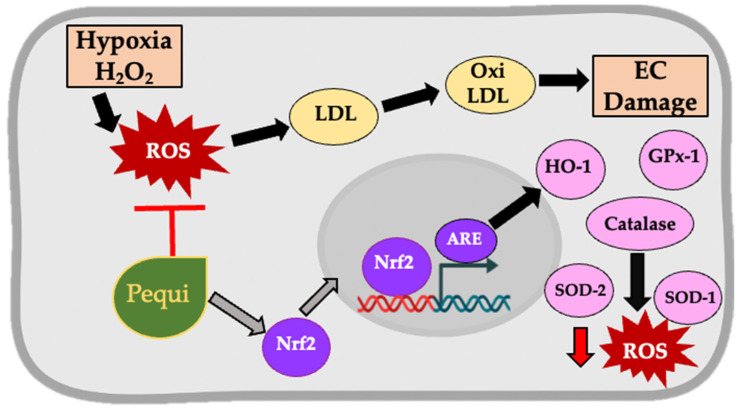
Proposed mechanisms of reduction in ROS in human coronary endothelial cells (HCAEC) by pequi extract. Hypoxia and H_2_O_2_ increase cytosolic and mitochondrial ROS in HCAEC. Polyphenol-rich pequi extract induces expression of antioxidant enzymes as a first line of defense against ROS, possibly by activating the Nrf2/ARE pathway and may reduce ROS and induce HCAEC proliferation. LDL: Low-density Lipoprotein; EC: Endothelial Cells; ARE: Antioxidant Responsive Element; Nrf-2: Nuclear factor-erythroid factor 2-related factor 2; HO-1: Heme Oxygenase-1; SOD-1: superoxide dismutase-1; SOD-2: superoxide dismutase-2; ROS: Reactive Oxygen Species.

One of the possible mechanisms involved in the ROS decrease by pequi extract is the activation of the nuclear factor (erythroid-derived 2)-like 2 (Nrf2)/antioxidant response element (ARE) pathway. There is compelling evidence that phenolic compounds, such as the components of pequi extract, activate the Nrf2 pathway and reduce ROS in various cells and tissues [33]. Nrf2/ARE reduces ROS by promoting the expression of antioxidant enzymes, such as heme oxygenase-1 (HO-1), glutathione peroxidase (GPx), and superoxide dismutase (SOD) [34]. These enzymes are significant players in maintaining redox balance within the cell [35], as reported here (Figure 6A–D). Since the expression levels of GPx, catalase, SOD-1, and SOD-2 are collectively increased by pequi, activation of the Nrf2 pathway may play a significant role in decreasing ROS levels in HCAEC reported here (Figure 7).

The reduction in ROS levels by pequi extract shown here may also decrease LDL oxidation. Oxidized LDL impairs the function of cardiovascular cells, including EC, a key mechanism leading to CVD and atherosclerosis [36]. Polyphenols from different sources have been reported to reduce LDL oxidation [37] or serum LDL levels, including the oil from the fruit pulp of pequi [38,39].

The addition of pequi extract to HCAEC significantly increased cell proliferation (Figure 5). Similarly, the addition of gallic-acid-rich leaf extracts of Toona sinensis, a plant commonly used in traditional Chinese medicine, reversed the decrease in cell viability by AAPH (2,2_-azo-bis(2-amidinopropane) hydrochloride), a water-soluble free radical generator [40]. The increase in endothelial cell proliferation due to pequi extract is substantial, since endothelial cell proliferation and migration are the key features of angiogenesis [41]. Another crucial evidence of the potential of the extract to protect against oxidative stress was the upregulation of antioxidant enzymes, particularly SOD-1 and -2, catalase, and glutathione peroxidase. Since these enzymes are the major players in maintaining redox balance within the cell [35], their collective increased expression may play a significant role in decreasing ROS levels in HCAEC.

## 5. Conclusions

The results presented in this study collectively demonstrate that the ethanolic extract of pequi fruit bark possesses a high capacity for scavenging free radicals in vitro, decreases cytosolic and mitochondrial ROS contents, and stimulates proliferation in HCAEC subject to stressful conditions such as hypoxia or exposure to H_2_O_2_. Ongoing studies in our lab will address the mechanisms by which pequi extract induces antioxidant signaling pathways and endothelial cell proliferation.

## Data Availability

The data is contained within this article.
